# Education strategies to facilitate lifestyle medicine practice within health systems: a multiple case study of US health systems

**DOI:** 10.1093/tbm/ibaf042

**Published:** 2025-09-14

**Authors:** Meghan L Ames, Samantha M Sundermeir, Kara L Staffier, Bruce Weeks, Melissa M Reznar, Tyler Hemmingson, Shannon Frattaroli, Joel Gittelsohn, Micaela C Karlsen

**Affiliations:** Department of International Health, Johns Hopkins Bloomberg School of Public Health, Baltimore, MD, United States; Department of Health Policy and Management, Johns Hopkins Bloomberg School of Public Health, Baltimore, MD, United States; Department of International Health, Johns Hopkins Bloomberg School of Public Health, Baltimore, MD, United States; American College of Lifestyle Medicine, Chesterfield, MO, United States; Department of Health Sciences, Oakland University, Rochester, MI, United States; American College of Lifestyle Medicine, Chesterfield, MO, United States; Department of Health Policy and Management, Johns Hopkins Bloomberg School of Public Health, Baltimore, MD, United States; Department of International Health, Johns Hopkins Bloomberg School of Public Health, Baltimore, MD, United States; American College of Lifestyle Medicine, Chesterfield, MO, United States; Applied Nutrition and Global Public Health, University of New England, Biddeford, ME, United States

**Keywords:** implementation science, qualitative research, lifestyle medicine, continuing medical education

## Abstract

**Background:**

Lifestyle medicine (LM) is an evidence-based field of medicine that is effective in treating and preventing leading causes of morbidity and mortality. Despite demonstrated impact, few physicians and other healthcare professionals regularly implement LM. Continuing education may be an effective avenue for improving practitioner knowledge, confidence, and practice of LM, but there is a gap in the understanding of how educational content and strategies can be utilized to effectively increase LM adoption. The purpose of this study is to identify educational strategies that facilitate the implementation of LM in health systems (HS).

**Methods:**

Eight US HSs participated in this multiple case study. We conducted in-depth, semi-structured interviews (*n* = 68 total; 6–8 within each HS) with HS employees leading and delivering LM programs. Interviews included questions about LM implementation and educational strategies. Transcripts were analyzed following the framework analysis approach. Strength of endorsement was assessed through quantitative and qualitative analysis.

**Results:**

Four topic areas were identified as critical content for effective continuing education in LM. The need for further education in behavior change counseling received the strongest endorsement. Other topics included LM definition and evidence, referral opportunities, and business development skills. Ten types of continuing educational strategies were identified that facilitate LM. There was the strongest endorsement for pilot programs, employee wellness, and interpersonal educational activities, including peer-learning, communities-of-practice, and supervisor-learning/mentorship.

**Conclusion:**

Continuing education can facilitate LM implementation in HSs. Educational strategies should emphasize training that builds skills in behavior change counseling, leverages employee wellness pilot programs, and nurtures interpersonal learning.

Implications

- **Practice:** Educational strategies designed to facilitate lifestyle medicine implementation must address behavior change counseling and nutrition and should include multiple modalities that emphasize interpersonal and experiential learning.
- **Policy:** Graduate medical education programs must include behavior change counseling and nutrition.
- **Research:** Future research is needed to identify enhancement activities that enable the most transfer of learning from educational interventions.

## Background

Lifestyle medicine (LM) is an evidence-based field of medicine demonstrated to be effective in treating, preventing, and improving outcomes associated with leading causes of morbidity and mortality, specifically cardiovascular disease [1, 2], type 2 diabetes [3, 4], and cancer [5–7]. It can be integrated into both primary and specialty care and builds upon behavior modification in six pillars, as identified by the American College of Lifestyle Medicine (ACLM): a whole-food, plant-predominant eating pattern; regular physical activity; restorative sleep; stress management; avoidance of risky substances; and positive social connection [8]. Despite demonstrated effectiveness, few physicians and other providers implement LM at optimal rates [9–13].

Implementation science can aid in understanding why healthcare professionals are not practicing lifestyle medicine. The Implementation Research Logic Model (IRLM) combines the implementation determinants (facilitators and barriers), implementation strategies, mechanisms of change, and implementation outcomes to illustrate a series of pathways that can advise implementation efforts [14]. When applied to LM, we can explore the determinants impacting adoption of LM practice by healthcare professionals and identify potential strategies for supporting LM adoption. Specifically in this study, we explore the inadequate LM knowledge, skills, and confidence as an implementation barrier, and LM education as an implementation strategy.

Lack of clinician skill and confidence in LM-related topics is a commonly noted implementation barrier [9, 12, 15–17]. In one study at an academic internal medicine residency program, 8 in 10 medical attendings (physicians) reported that physicians are not knowledgeable about cardiovascular disease prevention guidelines [9]. Another study found that less than half of primary care physicians reported high confidence, assisting patients to quit smoking [11].

Education is a well-supported implementation strategy to address lack of knowledge and confidence [17–20]. Professional continuing medical education (CME) and other continuing education serve the purpose of maintaining, enhancing, or establishing skills and knowledge needed by physicians and other healthcare professionals to provide medical care [21]. Implementation scientists identify training and education of stakeholders as one of nine clusters of implementation strategies identified by implementation science clinical practice experts [22]. Along the learning process, andragogy (adult learning) experts identify two distinct stages, which are illustrated in the Longitudinal Education for Advancing Practice (LEAP) model. First, the learning process is initiated by training, which focuses on specific knowledge and initial skill acquisition [23]. Following initial training strategies, learners must be supported by consultation, which can take the form of supervision, peer-learning (e.g. learning collaboratives), and coaching.

There is evidence that targeted educational interventions can improve participant knowledge, competency, and LM practice [17, 18]. However, additional research suggests that training alone is insufficient to change practice behavior, and healthcare professionals need additional support [24, 25]. More insight is needed to effectively design, disseminate, and enhance LM educational strategies that will increase practitioner implementation of LM.

To date, a gap exists in understanding how to effectively leverage educational strategies to facilitate practitioner adoption of LM practice. Specifically, we do not know which educational strategies health systems (HSs) are employing, how they are incorporating these strategies, and how various determinants influence the effectiveness of these strategies. To address these gaps, this research explored the following questions:

1. How is the selection or impact of educational strategies influenced by individual- and system-level determinants?
2. What educational strategies are commonly leveraged to facilitate LM implementation?
3. What are the mechanisms of change by which educational strategies facilitate LM implementation?

## Methods

### Research design

This study analyzed in-depth interview transcripts that were collected as a part of the *Lifestyle Medicine Integration in Health Systems: A Case Study Project.* This study follows an exploratory multiple-case study design [26] and is described in-depth elsewhere [27]. The unit of analysis is individual Hss, which are also referred to as “cases.”

### HS (case) recruitment

Between 3/21/22 and 4/1/22, we distributed recruitment messages via the ACLM Health Systems Council (HSC) emails, reaching approximately 150 HSs that offer LM programs. Fifteen HSs volunteered to participate in the study by submitting a self-nomination form. The form included information about HS location, patient population, payers, and the availability of LM programs and staff. HSs were asked to indicate whether LM was a “specialty” (stand-alone area of treatment programming) or a “sub-specialty,” indicating that LM is an adjunct approach embedded in other treatment specialties. The study team purposively selected an initial five health systems (HS) A–E that differed in size, geography, patient population, payer model, and age of the LM program [28]. After starting interviews with the initial sample, an additional two health systems (HS F–G) were selected, again to maximize variation. At this stage of sampling, the study team consulted the ACLM Health Systems Council staff coordinator to access more nuanced information regarding nominated health systems and advise on selection. The study team also acknowledged that a contrasting case would elucidate other insights and expand the generalizability of the findings [28]. To achieve this, they directly recruited an eighth health system (HS H) that had notably reduced LM programming and discontinued membership with the ACLM Health Systems Council.

### In-depth interviews

Health system liaisons provided a list of 8–20 employees that included health system leaders/administrators (including billing professionals), physicians, nurse practitioners, registered dietitians, behavioral health specialist/health coaches, exercise physiologist/physical therapist/kinesiologist, and mental health professionals. Study team members sought to conduct 6–8 in-depth interviews per case (health system), with priority given to interviewing health system leaders/administrators and physicians.

Interviews were conducted via video call (using Zoom Video Communications [29]), telephone, or in-person, and targeted to last 45–60 min. Interviews were open-ended and exploratory, and followed a semi-structured interview guide designed to allow for adaptability based on interviewee or health system context. Interview topics included history of the LM program, staffing and operations, patient population, each of the LM pillars, administrative or cultural supports and challenges, and educational strategies. The interview guide was loosely structured on the determinants section of the IRLM, which identifies five domains: intervention characteristics, inner setting, outer setting, characteristics of individuals, and process [14]. The complete interview guide is published elsewhere [30]. Minor updates to the interview guide were made as emergent topics were identified. If warranted, the study team requested a follow-up interview with an interviewee. Interviews were recorded via Zoom and transcribed verbatim.

### Data analysis

Interview transcripts were analyzed following the framework analysis approach [31] and using a two-cycle combination of deductive and inductive coding methodologies [32]. In the first cycle, data were coded using the Dedoose qualitative analysis software (version 9.0.107) [33]. The initial set of codes was deductively identified from adult learning literature and arranged to reflect the IRLM and the LEAP model [14, 23, 32]. Following coding of one-third of the transcripts, the primary analyst, MLA, reviewed code-related memos and revised the codebook. Revisions included merging of existing codes to reflect overlapping application of codes, addition of new codes where relevant content was not being captured, and removal of irrelevant or redundant codes. The primary analyst then conducted multiple iterations of cycle 2 coding to apply the final set of codes to all transcripts. The cycle 2 codebook is included in Appendix A, see online supplementary material.

**Figure 1:**
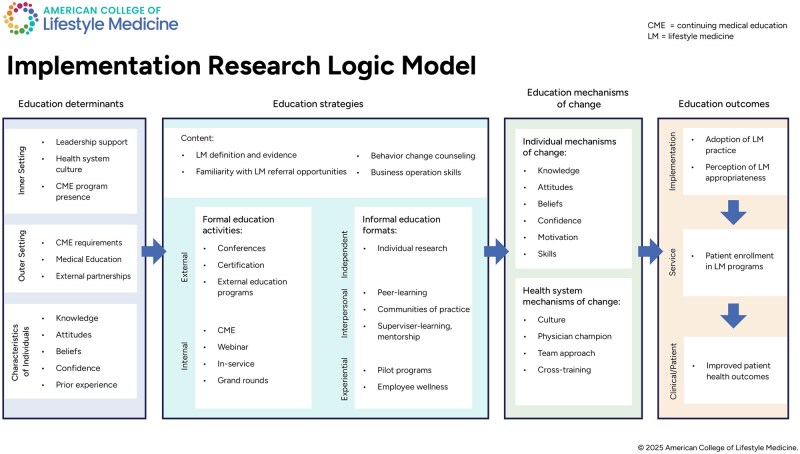
Generated themes of implementation determinants, strategies, mechanisms of change, and outcomes related to education interventions to promote LM practice. Adapted from the Implementation Research Logic Model [14]. CME, continuing medical education; LM, lifestyle medicine.

Following coding, the primary analyst reviewed the final codebook and analytic memos to identify thematic patterns. The results were summarized in an analytical framework, which was reviewed and approved by the study team [32, 34, 35]. The primary analyst used Microsoft Excel [36] to create a cross-case framework matrix based on the analytical framework and populated it with notable findings and conclusions (including examples and relevant quotes) for each site and construct. The entire study team then iteratively proposed, discussed, and reviewed findings and conclusions until consensus about explanatory themes was reached. To present the findings, the study team populated the IRLM with the generated themes (Fig. 1) [14].

During analysis, specific educational strategies were identified, and the strength of endorsement was assessed to determine strategies that were reported very frequently or had a great impact. Frequency was assessed by the number of cases endorsing each theme, reported in Table 1. Analysts also reviewed how many interviewees referenced each theme, how often they referenced it, and how they described it. All of this information was used to assess the strength of the endorsement for each strategy and theme.

**Table 1:** Summary of self-reported characteristics of participating health systems

Site code	Region	Level of LM focus^a^	Reach^b^
A	South	Sub-specialty	Small
B	West	Specialty	Large
C	Midwest	Sub-specialty	Medium
D	West	Specialty	Medium
E	South	Specialty	Medium
F	Midwest	Sub-specialty	Small
G	West	Specialty	Large
H	South	–	–

a “Specialty” indicates LM is a stand-alone area of treatment programming; “sub-specialty” indicates LM is an adjunct approach embedded in other treatment specialties.

b Reported estimated number of patients receiving LM care at time of nomination, where “large” >5000, “medium” is 1000-5000, and “small” <1000.

### Reflexivity

The study team was comprised of a diverse group of ACLM staff, graduate-level public health and medical students, and academic researchers who offer expertise in public health, healthcare management, lifestyle medicine, and qualitative research. The team was led by two researchers. One researcher, MCK, is the senior director of research at ACLM. The other researcher, JG, is a professor in public health with expertise in qualitative research and multiple case study methodologies.

M.L.A. and M.M.R. served as the primary and secondary analysts, respectively. M.L.A. is a registered dietitian with a background in clinical practice and program implementation. M.M.R. is a qualitative and quantitative researcher with a research focus on lifestyle behavior intervention delivery in medical and community settings. Both analysts take a constructivist approach to qualitative research, which acknowledges that the human experience is critical to all knowledge and insights [37].

## Results

Eight health systems (cases) participated in this multiple case study. Three reported that lifestyle medicine was a specialty of their health system, and four reported it was a sub-specialty (the datum for one site is missing). Additional health system information can be found in Table 1. Health System H did not complete the nomination form as it was directly recruited.

We conducted 68 interviews with 63 individuals. Follow-up interviews were completed with five individuals to answer specific clarifying questions, provide missing details, or explore a topic not previously discussed. At least two health system leaders/administrators and at least one physician were interviewed from each health system. Additional interview counts are included in Table 2. Generated themes are shown in an adapted IRLM in Fig. 1 and described below.

**Table 2:** Number of completed and invited interviewees at participating health systems

Health system	Health system leader/administrator	Physician	Dietitian	Behavioral health specialist/health coach	Nurse practitioner	Exercise physiologist/physical therapist/kinesiologist	Psychologist/psychiatrist	Other	Total
A	4/6^a^(1)	2/3	0/1	0/0	1/1	0/2	1/1	0/3	8/17^a^(1)
B	4/5	1/1^a^(1)	2/2	2/2	1/1	1/1	1/1	0/1	12/14^a^(1)
C	2/3^a^(1)	2/3	2/2	1/1	0/1	0/0	0/0	0/1	7/11^a^(1)
D	4/4	5/5	0/0	0/0	0/0	0/0	0/0	0/0	9/9
E	3/3	1/1	0/2	1/1	1/1	0/0	0/0	0/0	6/8
F	2/2^a^(1)	1/1^a^(1)	1/1	1/1	0/0	1/1	0/0	0/0	6/6^a^(2)
G	3/3	2/2	0/1	1/1	1/1	0/0	0/0	0/0	7/8
H	3/3	2/3	2/2	0/0	0/0	0/0	0/0	1/1	8/9
Total	25/29^a^(3)	16/19^a^(2)	7/11	6/6	4/5	2/4	2/2	1/6	63/82^a^(5)

Presented as “[number of completed interviews]/[total number of invited interviews]”.

a Indicates count of individuals for which a second interview was conducted.

### Determinants impacting LM educational strategies and outcomes

The study team identified 11 determinants that influenced the selection and implementation of educational strategies, as well as their impact on practitioner capability and likelihood to practice LM. These included knowledge, attitudes, beliefs, prior experience, leadership support, health system culture, CME program presence, CME requirements, medical education, and external partnerships. Observed determinants are described as either characteristics of individuals, inner-setting determinants, or outer-setting determinants, as defined in the IRLM [14, 30].

#### Characteristics of individuals (knowledge, attitudes, beliefs, confidence, prior experience)

The knowledge, attitudes, and beliefs of healthcare professionals were reported to influence educational strategies. Knowledge was often dependent on prior education and exposure to LM. Some interviewees noted a resistance among practitioners—specifically physicians—that stemmed from their formal medical education. One physician (HS H) stated, “Physicians, even till this day, if you go and look at medical school curriculum, there’s very little training in anything related to lifestyle medicine and specifically related to nutrition.” Others acknowledged challenges and at times resistance to attitudes in support of LM and the shifting mindset “to get away from the fee-for-service and really [look] at value-based care” (health system leader, HS E). As another health system leader (HS H) explained, “primary care providers can sometimes feel threatened [by LM]… in their minds they feel like they’re already doing it. And so helping to break down some of those barriers and those misconceptions…is key.”

Healthcare professionals who were enthusiastic about practicing LM often had a personal experience with LM that inspired them to learn more. For example, one interviewee described how being diagnosed with multiple sclerosis motivated them to read books and attend conferences about LM. They got involved with a small group of physicians who were also learning about LM, and shared their experiences with lifestyle interventions for treatment of their condition. Following personal success with a plant-based diet, this person advocated for more plant-based options for employees and patients and was a critical catalyst in the adoption and dissemination of LM at their health system. Similar anecdotes of personal experience were reported in seven health systems.

#### Inner-setting determinants (leadership support, health system culture, CME program presence)

Health system-level determinants influenced the selection and implementation of education strategies. Some health systems reported established practices of offering CME and grand rounds, which could serve as platforms for LM education. These groups often drew on internal expertise and were more likely to employ peer and supervisor-learning and mentorship strategies than other health systems that did not have such experts on staff. For these health systems, external partners, such as ACLM, PIVIO—the Complete Health Improvement Program (PIVIO) [38] and the Diabetes Prevention Program (DPP) [39] were engaged to train employees.

Leadership support and buy-in served as a catalyst for accessing additional resources, such as funding and staff time, and creating a culture supportive of LM practice. One health system (HS D) worked closely with ACLM to offer a 2-day virtual LM education program that prepared approximately 200 participants to achieve their LM certification.

#### Outer-setting determinants (CME requirements, medical education, external partnerships)

Because most clinical practice professions require certification that mandates CME, health systems commonly reported having a structure or resources to offer learning opportunities approved for continuing education by accrediting bodies. In some health systems, this existing CME structure was leveraged for LM education. About half of the health systems also reported leveraging partner relationships, such as with PIVIO, to offer LM training to employees. These types of training partnerships were more common among health systems that offered prioriatary wellness programs (e.g. PIVIO) as a part of their LM services.

### Content and format of educational strategies

The study team identified 4 critical topic areas and 10 types of educational strategies that were leveraged by some or all of the health systems. Table 3 includes descriptions, examples, and proposed mechanisms of change of these educational strategies. The greatest training need identified was in behavior change counseling skills. Strategies receiving the strongest endorsement included pilot programs, employee wellness initiatives, and interpersonal educational.

**Table 3:** Education strategies identified in health systems

Number^a^	Education strategy	Description	Examples	Mechanism of change	Other considerations
8	Supervisor-learning/mentorship	Formal or informal teaching or modeling of skills and practices	Physician leader hosts a cooking class for employees, who in turn share with patients	Individual KABC Institutional knowledge Leadership support Workplace culture	Must diversify leader/supervisor input to avoid burnout
8	Certification	Credential awarded by a national professional organization after recipient demonstrates achievement of specified competencies	Achievement of the ABLM board certification	Individual KABC Institutional knowledge Leadership support	Can be costly for many employees to be certified; may need additional practice opportunities to hone skills
8	Peer-learning	Skills, knowledge, or practice opportunity provided by a colleague	Employee with expertise in sleep health created an insomnia education class that provided other clinicians with the skills and knowledge to advise their own patients on sleep health	Individual KABC Institutional knowledge Workplace culture	Must ensure that peer instructors are adequately skilled and knowledgeable to teach others
8	Communities of practice	Structured engagement that allows organizations or colleagues to learn from one-another	A large health system establishes a LM practice group to review LM activities at various locations and share best practices	Individual KABC Institutional knowledge External partnerships Workplace culture	Some experiences may not be transferrable to other settings
7	CME/webinar/in-service/grand rounds	Learning strategies provided to medical professions and often required to maintain practice licensure	A unit on a health system hosts a lunch-and-learn book club where attendees review and discuss preparatory materials for the LM certification	Individual KABC Institutional knowledge Workplace culture (when completed as a team)	
6	External education programs	Educational programs delivered by private training organizations or trade associations, sometimes associated with certification, continuing education credits, or delivery of proprietary programs	A clinic supports a few senior clinicians to become Lifestyle Medicine Coaches, certified through Wellcoaches	Individual KABC Workplace culture (when completed as a team)	Added expense; may be required to implement proprietary programs
6	Conference attendance	Large, often geographical diverse, gatherings of professionals with a shared interest or practice area	Connecting with colleagues from different organizations at ACLM’s annual conference	Individual KABC External partnerships Leadership support	Added expense, especially if sending multiple staff
6	Independent research	Self-lead investigation via reading, attending lectures, or conducting other learning	An individual employee investigates LM in an effort to treat their own health condition	Individual KABC Institutional knowledge	
5	LM pilot program	Trial of an intervention in a limited context to assess feasibility and identify most effective strategies	A health system pilots PIVIO with a subset of employees and plan to expand it to patients also	Individual KABC Institutional knowledge Prior experience	Could be costly and not deliver results
5	Employee wellness program	Incorporation of LM activities into new or existing employee wellness programs	By piloting with employees, clinicians had first-hand experience with benefits of LM	Individual KABC Prior experience Workplace culture	Can be met with challenges when expanding to patient populations

a Number of health systems reporting strategy; strategies are listed from most to least commonly reported. KABC, knowledge, attitudes, beliefs, and confidence.

#### Content of educational strategies (LM definition and evidence, familiarity with LM referral opportunities, behavior change counseling, business operations skills)

Four topics emerged as being critical to LM education: LM definition and evidence; familiarity of LM programs at health system; behavior change theories and techniques; and business operations skills and knowledge.

Interviewees reported common misconceptions about what LM is and is not. One health system leader (HS D) noted that some physicians think LM is just “complementary medicine…[when] it is actually a way of supporting your patients.” Across all health systems, educational efforts emphasized the physical activity and nutrition pillars—although nutrition received stronger endorsement. This is described by a physician from HS D, “We focus mostly on what I call the primary two, which is healthy eating and exercise. And I think that is what makes sense to most people. And then we layered on the other four later.” Education on sleep health and stress management was described by four health systems, and social connectedness was described less frequently. Interviewees commonly reported that most substance-use services were offered in specialty clinics or programs.

Many educational strategies reported by interviewees sought to make healthcare professionals aware of the LM offerings, internal and external to their health system, to which they could refer patients. As one physician (HS D) noted, “it’s also important that the people you work with know about what existing resources are,” and that education must be ongoing because “you’ll always have new staff or new physicians joining.” This person went on to mention that they always speak about LM services during the orientation offered to new healthcare professionals at their health system.

Behavior change counseling received strong endorsement and was noted across all of the eight health systems as a topic for which more education was needed. Specific skills included assessing patient readiness to change, facilitating group counseling, and motivational interviewing. As one health system leader (HS B) noted, “We need more resources for health coaching opportunities, resources on motivational interviewing … all of those behavior change tactics that we know are going to be crucial for influencing our patients’ lives.”

Participating health systems also reported that healthcare professionals, especially health system leaders, needed additional education in business operations. They noted that many healthcare professionals did not have the business skills needed to launch a profitable LM operation, hindering their ability to be successful. As one physician (HS G) put it, “[our] belief of the power of lifestyle [medicine] … starts to very quickly erode when anybody tries to figure out how to operationally run a clinic, knowing that it’s not going to generate the revenue of an emergency room or an orthopedic clinic … The math is working against us.” A leader at HS D noted they had received business operations training as a part of a leadership program their employer sent them to. Although the training was not specific to the business of LM, the individual reported that they were able to apply many of the skills to the LM program they managed.

#### External formal education strategies (conferences, certification, and external education programs)

External educational sources, such as certification, conferences and educational programs, were commonly reported as resources used by health systems. They were particularly useful for health systems that did not yet have an established LM program, since often these were settings where expertise was not present internally.

Across all eight health systems, there were health professionals and physicians who had achieved LM certification, and this was one of the most-discussed resources. Interviewees from some health systems reported that achievement of certification by their physician champion was a critical catalyst in launching the LM program and often resulted in recruitment of others to pursue certification. One physician (HS H) participant responded that after getting LM certified, they “helped 15 other people, a few physicians and nurse practitioners and I think we had a PA, go through ACLM and get board certified.” At least two health systems established resources to support employees to achieve certification. In one health system, this was as simple as a book club where attendees reviewed the certification exam preparatory materials. Another health system coordinated a multi-day education program to prepare healthcare professionals to take and pass the LM certification exam. Although LM certification was reported to improve the credibility of healthcare professionals with patients and colleagues, some interviewees noted that certification was not essential to incorporate LM approaches into one’s practice.

Six of the participating health systems reported leveraging conference attendance for educational purposes. A commonly shared anecdote was that of a physician leader becoming “impassioned” about LM after attending an ACLM or Food Is Medicine conference, and “[bringing] information back to the community” (health system leader, HS E). Even among more advanced health systems, participation at LM conferences resulted in healthcare professionals learning about and implementing new practices, such as shared medical appointments, or programs, such as PIVIO [38]. Finally, conferences served as critical opportunities to create temporary communities-of-practice, discussed further below. This is exemplified in the anecdote shared by a physician (HS C), who noted that they felt encouraged when they met with a person from another health system during a workshop at the ACLM conference, were reassured that they were “doing this right.”

External educational strategies were noted by six of the eight health systems. ACLM, Food Is Medicine [40] and PIVIO [38] were most commonly reported as external education programs. Other education programs leveraged were developed by The Institute of Lifestyle Medicine [41], Wellcoaches [42], Physicians Committee for Responsible Medicine [43], Ornish Lifestyle Medicine program [44], Healthy Kitchens Healthy Lives [45], the American College of Sports Medicine [46], the Academy of Nutrition and Dietetics [47], and other health systems.

#### Internal formal education strategies (CME, webinar, in-service, grand rounds)

All health systems described internal formal education strategies that included in-services, grand rounds, and webinars. Some educational activities were approved for CME or other continuing education credits required by professional credentialing organizations. Some health systems had planning committees that organized internal education activities specifically related to LM. Others incorporated LM education into educational activities coordinated by the health system’s CME office.

These were delivered by a mix of external partners, such as those listed above, national experts, and internal experts. It was commonly described that a more experienced healthcare professional would deliver an education session to their colleagues. For example, one physician from HS A noted that she teaches LM to the residents and is hopeful that “at least they’ve been exposed to this way of thinking, and maybe they’ll think to ask their patients some of these questions, or think about sleep, or think about, you know some of these other pillars because they’ve seen it done.” Additionally, some aforementioned strategies to aid in LM certification preparation (such as the LM book club or multi-day education program) could also be considered internal formal education strategies.

#### Informal education strategies (independent, interpersonal, and experiential learning)

Participants described employing a wide variety of informal educational strategies to support LM implementation. These strategies can be broadly grouped into individual, interpersonal, and experiential learning strategies.

Individual learning strategies that were described included reading and attending or listening to live or recorded expert talks. Despite being reported in six of the eight cases, these strategies did not receive the same level of endorsement as others. Participants in independent learning strategies were often described by individuals who were pioneers in their health system and perhaps were not aware that there was a broader LM movement. Others who commonly discussed independent research were motivated by a personal health challenge or experience that they wanted to learn more about.

Interpersonal learning received strong endorsement and was emphasized by all participating health systems as a critical strategy to disseminate knowledge and transform cultures and environments. These included peer-learning, communities-of-practice, supervisor-learning, and mentorship. Supervisor-learning and mentorship was the most common educational strategy across all health systems. Peer-learning and commu­nities-of-practice were also reported by all health systems, although not as frequently.

Participants described learning from health system leaders who served as mentors and role models. One physician (HS D) mentioned their health system’s “see one do one teach one model,” explaining that their physician champion will “teach someone not only how to facilitate a culinary session, but, how to do the actual cooking, … [then they’ll] join her for one or two [sessions] … and then they’ll teach a few.” In some health systems, those individual leaders were providing one-on-one education or mentorship, and in other health systems, they offered a distal example of how to practice LM. One health system leader (HS H) described the “sizable personal transformation” they experienced as they learned about their physician champion’s vision for LM.

Peer-learning was commonly shared as a strategy for onboarding and cross-training staff to expand reach and services. As one physician (HS D) notes, “The secret sauce is having the other health professionals edifying them.” An exercise physiologist (HS B) described attending the sleep hygiene classes offered by their psychologist to “cross pollinate” and “see what [their colleague is] teaching.” Additionally, informal peer-learning served as a catalyst for the spread of information. One health system leader (HS C) described this in the context of “the tipping point,” concept noting that they could not afford to train all of their employees, so instead trained “5% carefully selected people” hoping the content would “ripple out to the whole.”

Interviewees also noted that clinical practice groups or communities-of-practice benefit LM implementation. Some of these groups were intentionally created, and others developed more organically. One physician (HS F) recalled how “simultaneously continuing to work with ACLM and the Health Systems Council has allowed again the opportunity to learn from the progress made by other health systems ahead of us.” Other participants described book clubs or monthly lunches that evolved out of formal education strategies and continued organically.

Experiential learning, via employee wellness pilot programs that incorporated LM, was reported by five of the eight health systems. However, for these five cases, this strategy was strongly endorsed and reported to be critical to the launch and growth of their LM program. Participants described that this served the dual purpose of: (i) exposing healthcare employees to LM practice and benefits; and (ii) providing a low-risk strategy for learning about operational considerations. One health system leader (HS B) describes learning from their pilot: “We sort of shot for the moon and developed this this very detailed program that frankly was overkill…after the program finished we realized it was not scalable in the current funding scheme.” These types of learnings were incredibly valuable to the health systems and enabled them to more effectively operate and scale their programs. Participants also reported that experiential learning was a good strategy to shift attitudes of healthcare professionals. One physician (HS F) describes that their colleagues’ completion of the PIVIO helped to “move the needle [on participant] understanding and awareness of lifestyle, medicine and whole-food plant-based diets.”

### Individual and health system mechanisms of change

When discussing educational strategies, study participants described how education impacted their LM practice. These mechanisms of change are reflected in Table 3 and explored below at both the individual and health system levels.

#### Individual-level mechanisms of change (knowledge, attitudes, beliefs, confidence, motivation, and skills)

Changes in healthcare professional knowledge, attitudes, beliefs, confidence, and motivation to practice LM were noted by most health system interviewees as a key output of LM education efforts. Participants described how LM education increased their confidence when speaking to patients and their knowledge about how to most effectively leverage health behavior change counseling to improve their patients’ health. Participants also noted benefits associated with LM certification. As one dietitian (HS B) put it, “I’m better able to see the importance of the other pillars [than] if I hadn’t done the certification…I love to say that I’m certified in Lifestyle Medicine. It kind of gives me credibility.”

#### Health system mechanisms of change (culture, physician champion, team approach, cross-training)

Mechanisms of change at the health system level included cultural shifts, reinforcement of a team approach, and cross-training. This was especially true when the educational experiences were shared among healthcare professionals, such as when clinics participated in educational strategies together. Participants described the value of “having a shared language” and a coordinated approach among specialties. For example, one health system sent a group of healthcare professionals to a coaching program. In response, one health system leader (HS B) noted, “we all we wanted to be able to speak a common language to the patients … [and] have a consistent approach.” They described how being familiar with the practices offered by their colleagues allowed them to offer complementary care. This was true with HS C that trained all their mental health providers in mind-body techniques for use in their practice to build upon the yoga offered by the exercise physiologists. Cross-training and coordinated care made providers feel like they were part of a team and allowed them to “practice at the top of their licensure.” (health system leader, HS G) One physician (HS B) described that investing in a multidisciplinary team “builds morale for your providers” and describes themself to be “an extension of the primary care providers and the sub-specialists,” noting the LM team is “giving care that they [primary care providers and sub-specialists] don’t have time to give.” Finally, four of the participating health systems reported that the role of the physician champion was critical. One physician (HS G) described, “I think that you have to walk the walk…”

In contrast, interviewees from the contrasting case (HS, H; where LM investments had been scaled back) described a lack of leadership support for LM that resulted in burnout and attrition of LM health professionals. One prior physician from HS H described that it “is really a shame … [that their colleagues] went through the struggles of getting board certified [in LM] … [and] why the health system doesn’t promote that is beyond [their] comprehension.” The lack of recognition and resources continued to strain employees, and ultimately resulted in many LM professionals resigning. A previous health system leader (HS H) who managed the LM offerings described realizing that broader health system leadership “just [didn’t] get it [speaking of LM] … It’s not how they’re trained. It’s not how they’re paid.” They described the resources offered to LM as “trying to strap wings on the Titanic with duct tape,” and eventually went on to leave the health system. Employee attrition and lack of leadership support eventually led to a dwarfing of LM programs at this health system.

## Discussion

This is the first study to explore education strategies for lifestyle medicine implementation in health systems. The analysis yielded a host of individual (e.g. knowledge), inner-setting (e.g. leadership support), and outer-setting (e.g. CME requirements) determinants influencing selection and adoption of educational strategies. Similarly, mechanisms of change were also identified at the individual (e.g. knowledge or motivation) and inner-­setting (e.g. culture) levels. Results identified four critical content areas and ten educational strategies that were leveraged by health systems to support LM implementation. Content areas included: LM definition and evidence; LM referral opportunities; behavior change counseling; and business operations. Behavior change counseling skills emerged as a topic of greatest training need. Strategies included: conferences; certification; external education programs; CME; webinars, in-service, and grand-rounds; independent research; peer-learning; communities-of-practice; supervisor-learning/mentorship; pilot programs; and employee wellness. Analysis revealed the strongest endorsement of pilot programs, employee wellness, and interpersonal educational strategies—including peer-learning, communities-of-practice, and supervisor-learning/mentorship. A comprehensive framework of these themes and their relationships is included in Fig. 1.

The critical need for skill building in behavior change counseling (especially for dietary behaviors), including motivational interviewing, was strongly endorsed across cases and interviewees. Motivational interviewing is a patient-led counseling approach that is a cornerstone of LM and has demonstrated effectiveness for behavior change [48–51]. This finding aligns with other research that demonstrates gaps in training at medical schools, through healthcare professional education and confidence in nutritional counseling. One study found that almost three-quarters of medical schools are not meeting the recommended 25 hours of nutrition education [52]. Another study determined that only half of medical residents reported feeling confident in their ability to support patients’ lifestyle behavior changes [16]. The American Heart Association acknowledges this need and calls for practice-based learning that incorporates behavioral and social sciences learnings [53]. Effective preparation of healthcare professionals in motivational interviewing has been demonstrated. Most successful interventions include intensive curriculum and opportunities for practice [54, 55].

This study revealed the critical role of employee wellness pilot programs in initiating and growing LM practice. Although it was not reported in all of the health systems, this strategy was important for the health systems that utilized it. Study participants reported two benefits. First, healthcare professionals are exposed to LM practices as participants in wellness programs, which is thought to improve knowledge and attitudes about and potential adoption of LM practice. The link between wellness program participation and LM attitudes has not been studied, but there is substantial evidence that supports an indirect path by which wellness programs improve healthcare professionals’ health behaviors [56–59], and healthcare professionals with healthier lifestyles are more likely to counsel health behaviors [12, 60, 61]. The second benefit of employee wellness program pilots is that LM program managers gain valuable insights to guide operational success. Similar benefits have been documented in other studies, showing the benefit of trialing and adapting specific strategies prior to broad dissemination [62, 63]. Additionally, the use of pilots or “staged implementation scale up” are widely recognized in the field of implementation science as implementation strategies [22, 64]. A third added benefit of this strategy is the resulting improvements in employee health and alignment with recommendations to institutionalize wellbeing [65].

This study highlights the importance of interpersonal learning strategies to facilitate and encourage LM practice in healthcare systems. The ability of short, didactic interventions to improve knowledge and performance of targeted content or skills is well demonstrated [66–68]. However, sustained changes in practice behaviors often require additional support or consultation [24, 25, 69, 70]. Supervisor-learning and mentorship was one of the most discussed educational strategies in this study, followed closely by peer-learning and communities-of-practice. This supports the findings of other research, suggesting that interpersonal connectedness with one’s educator has been demonstrated to impact educational outcomes [23, 71]. The need for strong cultural support for learning was further emphasized by the contrasting case (HS H), which experienced a substantial reduction in LM services after a shift in leadership and decline in LM support led to employee burnout and attrition.

This study is limited to educational strategies and does not explore additional implementation strategies, such as leveraging alternative payment schemas or preparing patients or consumers to actively participate [64]. Although healthcare professionals can be trained on how to conduct behavioral counseling, patient visits may still be too brief for effective counseling [72]. Educational strategies are likely necessary but not sufficient for broad adoption of effective LM practice. We can expect educational strategies to impact what conversations providers have with patients, the efforts they make to create and work with an interdisciplinary team, and, if in a position of leadership, the health system policies they can enact. To achieve broad changes, however, implementation strategies must address system level policies and processes, such as the use of shared-medical appointments and physician incentives.

Additionally, the results are subject to sampling bias, as all of the eight participating health systems had already initiated some type of LM program. HS H offers a contrasting case, but does not yield insights into health systems that are entirely naïve to LM. Thus, these findings should be translated cautiously, if at all, to health systems with no LM experience. Findings may also not be transferrable to all health systems that offer LM programs, since the eight participating health systems are not reflective of a representative sample. The study is strengthened by the use of implementation science frameworks (IRLM) and andragogy theories (LEAP) [14, 23, 30]. It includes an evaluation of mechanisms of change, which offers a richer understanding of the findings. A strength of our approach is that we used three different forms of triangulation: source/stakeholder (eight different types of professional roles), theoretical, and cases (eight different health systems).

## Conclusion

This study identified educational strategies that can facilitate LM implementation in health systems and, thus, may potentially contribute to reducing morbidity and mortality among patients. Educational strategies should emphasize education that builds skills in knowledge of nutrition and behavior change counseling, leverages employee wellness pilot programs, and nurtures interpersonal learning. Future research should investigate the lasting impact of these educational strategies and quantify the impact of LM education in health systems on patient outcomes.

## Supplementary Material

ibaf042_Supplementary_Data

## Data Availability

Data cannot be shared for ethical/privacy reasons.

## Welfare of Animals

This article does not contain any studies with animals performed by any of the authors.
