# Supplementary material for: Education strategies to facilitate lifestyle medicine practice within health systems: a multiple case study of US health systems
Source: Transl Behav Med. 2025 Sep 14;15(1):ibaf042. doi: 10.1093/tbm/ibaf042 (PMC12448416; doi:10.1093/tbm/ibaf042)
Supplement: ibaf042_Supplementary_Data [file ibaf042_supplementary_data.docx]

Appendix A. Cycle 2 codebook

| Parent code | Parent code modifier | Child code level 1 | Child code level 2 | Description |
| --- | --- | --- | --- | --- |
| factors | individual | - | - | determining factors, such as mindsets, norms, interests, and affiliations, that occur at the individual level |
| factors | individual | formal training | - | mention of if and how formal education (including medical education) or training (including internships or residency) impacted one’s preparation to practice LM |
| factors | individual | LM exposure | - | individual exposure to the practice of LM through professional experiences, but not through formal training or personal experiences |
| factors | individual | personal experiences |  | discussion of prior personal or non-professional experiences in the topics related to LM |
| factors | system | - |  | determining factors, such as structural characteristics, networks, culture, climate, and readiness, that occur at the health system level |
| factors | system | culture | - | cultural trends among health system employees that impact LM implementation |
| factors | system | leadership | - | health system leadership or policies that impact LM implementation |
| factors | system | partners | - | clinician training content related to what is LM and how to refer to it; similar to LM promotion |
| factors | system | space/ financial/ time resources |  | resources such as physical meeting space, funding, and staff time that impact LM implementation |
| mechanisms | individual | - |  | pathways by which an individual changes their LM implementation due to an educational practice |
| mechanisms | individual | practice | - | changes in a clinician's LM practice behaviors |
| mechanisms | individual | knowledge | - | changes in a clinician’s knowledge about LM that result from a training activity |
| mechanisms | individual | self-efficacy | - | changes in a clinician’s confidence or perceived credibility about LM that result from a training activity |
| mechanisms | individual | skill | - | acquisition or application of a new skill |
| mechanisms | system | - | - | pathways by which a system changes their LM implementation due to an educational practice |
| mechanisms | system | cross-training | - | formal or informal education of a clinician to support or compliment area of expertise of another clinician |
| mechanisms | system | growth | - | a LM program being able to increase the number of patients reached or diversify the types of programs offered |
| mechanisms | system | team approach | - | description of when different types of practitioners create LM synergy by combining their various expertise |
| mechanisms | system | culture | - | changes to culture, practice, camaraderie resulting from a training activity |
| training content | - | - | - | topics described by participants as being included in LM educational efforts or needed to be included |
| training content | - | behavior change | - | training content related to behavior change; could include counseling, motivational interviewing, theories of behavior change, etc. |
| training content | - | operations | - | clinician training content related to the business and operational considerations of creating a financial viable clinic |
| training content | - | referral | - | clinician training content related to what is LM and how to refer to it; similar to LM promotion |
| training content | - | LM pillars/ definition/ evidence | - | foundational definition of and evidence for LM; including of the six pillars |
| training format | formal | - | - | training strategies that are specifically funded, coordinated, or sponsored through a health-system program or effort |
| training format | formal | certification | - | mention of LM or other related certification (such as being certified as a diabetes care and education specialist) |
| training format | formal | CME | - | training or activity that meets requirements for licensure or certification |
| training format | formal | conference | - | synchronous in-person training; often lasting longer than one hour |
| training format | formal | in-service | - | mandatory training offered to employees during the workday |
| training format | formal | webinar/lecture | - | organized talk/lecture that is usually one-directional |
| training format | informal | - | - | training strategies that are not specifically funded or coordinated through a health-system program or effort |
| training format | informal | experiential | employee wellness | wellness programs directed at employees that enhance clinicians’ ability to offer LM by facilitating individual personal experience with LM, understanding of LM, appreciation for LM, and/or ability to deliver LM |
| training format | informal | experiential | learn by doing | respondent describes a pilot program and trial experience that helped them learn, practice, or solidify a knowledge or skill |
| training format | informal | experiential | pilot | enhanced ability to deliver LM by testing and revising programs/offerings/practices on a subset of patients/customers; mention of critical skill/confidence/knowledge that was gained through pilots or practice |
| training format | informal | individual research | - | self-directed research through reading, attending workshops/lectures, or speaking with experts |
| training format | informal | interpersonal | communities of practice | groups of peers with similar practice areas who discuss LM content and practice |
| training format | informal | interpersonal | mentorship | a professional relationship (could be a formal or informal) relationship that is established between two individuals with the purpose of advancing one’s knowledge, practice, confidence, strategy. |
| training format | informal | interpersonal | peer learning | formal or informal learning that takes place by a peer or colleague |
| training format | informal | interpersonal | supervisor learning | formal or informal teaching and learning from a supervisor or leader to a more junior employee |
